# The role of the hippocampus in flexible cognition and social behavior

**DOI:** 10.3389/fnhum.2014.00742

**Published:** 2014-09-30

**Authors:** Rachael D. Rubin, Patrick D. Watson, Melissa C. Duff, Neal J. Cohen

**Affiliations:** ^1^Department of Psychology and Beckman Institute, University of Illinois at Urbana-ChampaignUrbana, IL, USA; ^2^Department of Communication Sciences and Disorders, University of IowaIowa City, IA, USA; ^3^Department of Neurology, Division of Behavioral Neurology and Cognitive Neuroscience, University of IowaIowa City, IA, USA

**Keywords:** hippocampus, flexible cognition, social behavior, relational memory, amnesia

## Abstract

Successful behavior requires actively acquiring and representing information about the environment and people, and manipulating and using those acquired representations flexibly to optimally act in and on the world. The frontal lobes have figured prominently in most accounts of flexible or goal-directed behavior, as evidenced by often-reported behavioral inflexibility in individuals with frontal lobe dysfunction. Here, we propose that the hippocampus also plays a critical role by forming and reconstructing relational memory representations that underlie flexible cognition and social behavior. There is mounting evidence that damage to the hippocampus can produce inflexible and maladaptive behavior when such behavior places high demands on the generation, recombination, and flexible use of information. This is seen in abilities as diverse as memory, navigation, exploration, imagination, creativity, decision-making, character judgments, establishing and maintaining social bonds, empathy, social discourse, and language use. Thus, the hippocampus, together with its extensive interconnections with other neural systems, supports the flexible use of information in general. Further, we suggest that this understanding has important clinical implications. Hippocampal abnormalities can produce profound deficits in real-world situations, which typically place high demands on the flexible use of information, but are not always obvious on diagnostic tools tuned to frontal lobe function. This review documents the role of the hippocampus in supporting flexible representations and aims to expand our understanding of the dynamic networks that operate as we move through and create meaning of our world.

## Overview

“The measure of intelligence is the ability to change.”

-Albert Einstein

Humans are active agents in the world, constantly acquiring information about their environment, manipulating those representations, and synthesizing optimal behavioral and cognitive strategies to modify the world around them. This ability to flexibly employ different strategies is usually attributed to executive function and working memory systems supported by the prefrontal cortex (PFC). However, we suggest that in everyday, ecologically valid scenarios such flexible cognition places great demands on memory beyond what can be supported by PFC-associated working memory, drawing heavily upon memory representations that capture past experiences to inform future behaviors and decisions. Accordingly, we use the term flexible cognition to describe the adaptive process of generating, updating, modifying, and integrating past and present information in response to the demands and constraints of both the real-world environment and the experimental task, and aim to show its reliance on the hippocampal network. Thus, we suggest that the hippocampus, which has traditionally been associated with long-term, declarative, or episodic memory, is actually essential to the flexible cognition network whenever representations must be appropriately constructed, manipulated, and updated to respond to the task at hand, and reflect the social and environmental context.

We also note that the contribution of the hippocampus to flexible cognition is perhaps most apparent in the complex dynamics of social interactions. In everyday social interactions, subtle contextual differences (e.g., a single prior interaction with an individual) require extensive and flexible modifications of our behavior, driving us to select different words, draw upon shared knowledge, or use entirely different language and social conventions for interaction. For example, successfully navigating a dinner party requires making appropriate responses to both novel and familiar guests and updating representations of ongoing conversations. The ability to do so relies on information about the situation no longer in current sensory experience (e.g., “Who arrived on time?”), predictions based on prior knowledge (e.g., “What does Jen want to drink tonight?”), inferences based on existing relationships (e.g., “Does Hillary know Debbie?”), and much more. Hence, we suggest that rather than relying on memory processes associated with PFC networks that include executive and working memory functions, successful behavior increasingly depends upon the constant encoding, updating, and flexible manipulation of relational memory representations supported by the hippocampus. Otherwise behavior is driven by inappropriate, inflexible, and stereotypical behaviors guided by general knowledge (e.g., pour wine for everyone, regardless of an individual’s preferences).

In this review, we describe findings from studies that utilize a variety of cognitive neuroscience methodologies to elucidate the role of the hippocampus in flexible cognition and social behavior. We place particular emphasis on findings from patients with impairments resulting from hippocampal damage that establish the critical role of the hippocampus in a broad range of behaviors that require the flexible use of information. These findings provide unique insight into the nature and time course of the contribution of the hippocampus to flexible cognition across everyday tasks and social interactions. These data show that (1) the hippocampus is a critical component of the large network of brain structures implementing flexible cognition, and as a result (2) hippocampal-dependent representations are necessarily employed in situations requiring the flexible use of information. In particular, the hippocampus is critical for performance in complex and ecologically valid situations that unfold over time and involve dynamically binding together various pieces of information.

Disruptions in flexible cognition that result from hippocampal damage, however, do not always appear on neuropsychological tests of cognitive flexibility constructed to specifically measure either executive functions or more traditional forms of declarative memory. Thus, in later sections we outline implications for the inclusion of the hippocampus in the neural network supporting flexible cognition and discuss implications for future research, clinical practice, and re-conceptualizing the relationship between disorders of the brain and complex behavior. Understanding how the hippocampus contributes to adaptive behaviors necessary for navigating complex environments and social interactions is critical for clinicians seeking to understand the everyday challenges that patients with memory deficits face, and for investigators seeking to understand the relative contributions of different brain systems during all kinds of flexible cognition.

## The network of brain structures supporting flexible cognition and the hippocampus

Flexible cognition is often discussed in the context of executive function, supporting the ability to switch between competing goals, as well as contributing to high-level human behavior, such as planning, organizing, and decision-making (e.g., Eslinger and Grattan, 1993; Eslinger, 1996; Jurado and Rosselli, 2007). On neuropsychological assessments that rely on executive functions, patients with damage to frontal areas demonstrate impairments, exhibiting various forms of behavioral inflexibility that include perseveration of behavior, rigid rule structure, and social inappropriateness (e.g., although see Anderson et al., 1991; Lezak, 1993; Stuss and Alexander, 2000; Stuss and Levine, 2002; Alvarez and Emory, 2006). Thus, the frontal lobes are properly emphasized as making a critical contribution to flexible cognition and social behavior.

The frontal lobes, however, are part of a large and distributed network of brain structures that support the flexible use of information. For example, complex social interactions that rely on the flexible use of information involve various parts of the frontal lobes (e.g., medial, dorsolateral, orbital, and ventromedial prefrontal cortex), as well as structures located in temporal, parietal, and limbic circuits (e.g., superior temporal sulcus, amygdala, insula, somatosensory cortex, temporoparietal junction) (Adolphs, 2003; Hari and Kujala, 2009). Thus, there is consensus that complex information processing draws upon a variety of brain networks in order to respond to varying task demands; however, the usual description of the network for flexible cognition rarely, if ever, includes the hippocampus.

The omission of the hippocampus in descriptions of the flexible cognition network likely results from the strong association between the hippocampus and long-term memory. Traditional neuropsychological and laboratory tasks were designed to obtain process-pure measures that *distinguished* between executive function and memory abilities, rather than elucidated interactions between these functions. Yet, outside of the lab, everyday situations necessitate active engagement with the environment and other social agents. In these real-life situations, memory and executive function must interact seamlessly, and obligatorily, to meet the demands of a constantly changing environment. The demand on memory is particularly clear in social interactions that often require learning by observing others in similar situations, recognizing the shifting or changing status of friends and enemies, using language to communicate and re-describe events from multiple points of view, and imagining things that might happen to us in the future. These abilities require integrating information across multiple timeframes that may stretch from the distant past, to the present moment, to possible futures (Lemke, 2000; Adolphs, 2003; Cacioppo et al., 2006). That is, these abilities require representing information, such as previous conversations, alternate perspectives, shared and unshared experiences, and even fictive material that are not necessarily contained within the present moment or within the span of working memory. Therefore, the constant encoding, updating, and flexible expression of relational memory representations are required for flexible cognition, which depends heavily upon the hippocampal-dependent memory system.

## The hippocampus supports flexible cognition through the encoding and flexible expression of relational memory representations

Early neuropsychological studies in patients with hippocampal amnesia provided crucial insight into the organization of human memory and its instantiation in the brain, such that damage to the hippocampus and related medial temporal lobe (MTL) structures resulted in a profound but circumscribed amnesia (e.g., Scoville and Milner, 1957; Cohen and Eichenbaum, 1993). The memory system selectively affected in amnesia, and critically dependent on the hippocampus, is declarative memory (Cohen and Squire, 1980; Squire, 1992; Cohen and Eichenbaum, 1993; Gabrieli, 1998; Eichenbaum and Cohen, 2001). This form of memory represents information about the co-occurrences of people, places, and things, along with the spatial, temporal, and interactional relations among them, which often include personal awareness and social context, that constitute the autobiographical record of our lives (Cohen and Eichenbaum, 1993; Eichenbaum and Cohen, 2001). That is, the hippocampus is essential for representing the elements of everyday interactions and the relations among them, whereas surrounding MTL structures, the perirhinal cortex and the parahippocampal cortex, are characterized by the ability to support item (i.e., inflexible configural relations) and context memory, respectively (e.g., Cohen et al., 1997; Davachi, 2006; Eichenbaum et al., 2007; Ranganath, 2010b; but see Squire et al., 2007). The critical role of the hippocampus in relational representations has received considerable support in recent years (Davachi, 2006; Henke, 2010; Ranganath, 2010a; Olsen et al., 2012; Yonelinas, 2013).

Relational representations supported by the hippocampus are characterized by two hallmark features: (1) the *binding* of arbitrary relations between the elements of experience into durable representations of past experiences; and (2) the *flexible* expression of these representations, which allow for the search, reconstruction, and recombination of the information contained within them (as opposed to a “video-camera”-like recapitulation of prior events). This representational flexibility permits information to be searched and accessed across processing systems (e.g., when a rich, multisensory autobiographical memory is evoked by the sight of a familiar face or sound of a familiar song) and to be used in novel situations (e.g., when exploring a new environment or meeting a new person). Furthermore, the contribution of the hippocampus to relational representations need not be limited to the explicit awareness and retention of memory over long-term delays (Ryan et al., 2000; Eichenbaum and Cohen, 2001; Henke, 2010; Olsen et al., 2012). This conceptualization has implications for the involvement of the hippocampus during tasks on the time-scale of short-term or working memory, and outside the memory domain, when relational representations are required. We discuss these points in more detail later on.

The flexible nature of relational memory representations also makes contact with a long memory literature that presents memory as a flexible reconstruction of past events (Bartlett, 1932). This literature is frequently framed negatively, being primarily concerned with the study of memory’s imperfect accuracy (Neisser, 1982), such as the imperfect accuracy of eyewitness testimony (Loftus et al., 2008), or outright “false” memories (Loftus and Pickrell, 1995). However, the relational memory framework suggets that it is this same flexible reconstruction that enables us to update and integrate the information from previous experiences to other episodes and to generate new ideas. That is, binding and re-binding the individual elements of experience compositionally permits the encoding for time- and place-specific autobiographical experiences, as well as the representations of the relationships among *different* experiences which are impossible to appreciate *a priori* (Cohen and Eichenbaum, 1993; Cohen et al., 1997; Ryan et al., 2000; Eichenbaum and Cohen, 2001; Giovanello et al., 2003; Davachi, 2006; Eichenbaum et al., 2007; Konkel et al., 2008; Staresina and Davachi, 2009; Ranganath, 2010b; Olsen et al., 2012; Yonelinas, 2013). These hippocampal representations provide the basis for the larger record of one’s life, and as we emphasize, support the ability to adapt to changing circumstances and engage in complex social interactions, which are necessary for functioning successfully in the real-world. The flexible construction and use of these representations also implies a persistent need for memory search, updating, and transformation of previously encoded information, especially in contexts that require the tracking of multiple objects, locations, times, and individuals, embedded in diverse environmental and social contexts. The involvement of hippocampus in supporting interactions between diverse and complex elements required for cognitive and social abilities is well documented (O’Keefe and Nadel, 1978; Cohen, 1984; Squire, 1992; Cohen and Eichenbaum, 1993; Bunsey and Eichenbaum, 1996; Dusek and Eichenbaum, 1997; Eichenbaum and Cohen, 2001).

As mentioned previously, the relational memory framework suggests that the characteristic processing features of the hippocampus, the ability to bind together arbitrary relations and to support their flexible expression, occur independent of timescale. That is, whether the representations are being accessed on the timescale of long-term or episodic memory, short-term or working memory, or even during moment-to-moment processing. Recent findings support this idea: when tasks are constructed to require relational binding and representational flexibility, patients with hippocampal amnesia demonstrate impairments across minimal delays, and even when all the necessary information to perform the task is perceptually available (Hannula et al., 2006; Olson et al., 2006a,b; Barense et al., 2007; Warren et al., 2011; Watson et al., 2013). For example, we have shown that patients with hippocampal amnesia are impaired relative to matched comparison participants at forming both spatial and non-spatial relations among co-occurring items (e.g., the elements of furniture in a room and a face superimposed on a scene) at very short delays that are considered to be on the time scale of working or short-term memory (Hannula et al., 2006). Consistent with these findings, evidence from functional neuroimaging reveals hippocampal activations for relational information during these same short delays (Ranganath and D’Esposito, 2001; Hannula and Ranganath, 2008).

In light of this evidence, others are also exploring hippocampal contributions to formatting, updating, and actively using models of our experiences in navigating our world, social interactions and relationships (see Spreng, 2013 for introduction to Research Topic “Examining the role of memory in social cognition”). In the next section, we examine the contribution of the hippocampus to flexible and adaptive behavior, and the importance of the hippocampus to increasingly ecologically valid tasks that require flexible representations, whether those representations pertain to remembered events, or supporting online social, linguistic, or cognitive processing.

## The hippocampus and flexible memory representations are critical in many cognitive abilities and complex social behaviors

We suggest that the flexibility afforded by hippocampal representations permits various pieces of information to be called upon promiscuously to support diverse and complex cognitive and social abilities. The importance of flexible representations in many cognitive and social behaviors has recently been explored in a number of experimental paradigms in patients with hippocampal amnesia. These paradigms assess the ability of humans with hippocampal damage to perform tasks that approximate real-world interactions in which there is a high demand on flexible representations for adaptive and successful performance. The performance of humans with hippocampal damage on these tasks provides useful insight into the specific role that the hippocampus performs in supporting the flexible use of information. Indeed, we highlight a variety of findings from patients with hippocampal amnesia on both tasks in the cognitive and social domains, in which the basic processing mechanisms are not impaired (e.g., basic linguistic abilities are intact as patients with amnesia do not have aphasia), but the nature of the task places significant processing demands on the flexible use of information (e.g., using language flexibly to reflect changes in context or perspective during social discourse), resulting in abnormal or impaired performance. Thus, while the PFC may be important for switching between or integrating abstracted representations, the hippocampus is required to form and deploy those representations flexibly for use by other neural systems.

### Spatial navigation and active exploration of the environment

As we navigate and engage with our world, we are constantly, automatically, and obligatorily encoding relations (spatial or otherwise), updating mental representations, and using that information in real-time to guide our behavior. The contribution of the hippocampus to spatial information and navigation has an extensive basis in the literature stemming from early evidence of location-modulated cells in the rodent hippocampus (for review, see Burgess et al., 2002). Evidence suggesting the hippocampus is important for spatial navigation also comes from patients with hippocampal amnesia (Maguire et al., 2006), as well as findings from functional neuroimaging studies (Ghaem et al., 1997; Maguire et al., 1997, 1998; Hartley et al., 2003; Kumaran and Maguire, 2005; Spiers and Maguire, 2006), especially when successful navigation requires access to detailed spatial representations of recently learned information. For example, Maguire et al. (2006) examined the involvement of the hippocampus in navigating an environment learned long ago in a taxi driver with hippocampal amnesia. While performance was relatively intact on general orientation in the city, knowledge of landmarks and their spatial relationships, and active navigation along some routes, hippocampal damage disrupted the ability to navigate complex environments that required the use of roads that were not major arties in the city, even though the information had been learned prior to the onset of amnesia. These findings are broadly consistent with a theory of hippocampal processing emphasizing the flexible and dynamic use of information, since representing spatial information requires constructing and maintaining relationships between different elements in the environment, establishing maps, layouts, and spatially arranged compositions of elements. Once such a configuration has been encoded (such as the relationships between the buildings and streets that make up the layout of a city, or the hallways and rooms that make up the layout of one’s own home), we must continually update our own position as we move through the map, and compare this location with the desired destination. These elements are intrinsic to spatial navigation and place a great demand on the flexible information supported by the hippocampus (Eichenbaum et al., 1999; Eichenbaum and Cohen, 2001).

Even outside the realm of navigation, the ability to tailor our behavior to meet current situational demands and incorporate immediate sensory input to guide upcoming actions and choices relies on contributions from both memory and executive control systems (Squire and Zola-Morgan, 1991; Smith and Jonides, 1999; Eichenbaum and Cohen, 2001; Tanji and Hoshi, 2008). Recent neuroimaging research suggests that the hippocampus and areas of the frontal cortex, including dorsolateral PFC, support active exploration of the environment and may lead to optimization of behavior for learning and memory of new information (Voss et al., 2011a,b). Consistent with these findings, the benefit of active control during learning is absent in patients with hippocampal damage, suggesting the hippocampus may actually be a critical component of the network that supports such behaviors (Voss et al., 2011a). In this task, patients with hippocampal amnesia studied an array of common objects arranged on a grid and viewed one object at a time through a small moving windows. When the patients with amnesia were tested for memory of the items and their spatial layout, their performance did not improve, and was actually worse, when they had active control of the moving window during the study portion of the task. Research in hippocampal amnesia also suggests the hippocampus has an active role in acquiring information about the environment and using that information during ongoing processing to guide what information should be obtained next based on previous experience (Voss et al., 2011a,b; Yee et al., 2014). Together, these findings suggest that actively learning about the environment optimizes interactions among specialized neural systems and relies critically on the involvement of the hippocampus. Furthermore, the contribution of the hippocampus is far more immediate than would be suggested by traditional descriptions of hippocampal function that are limited to long-term memory. Thus, the contribution of the hippocampus stems from the fundamental role of the hippocampus in the flexible use of relational representations.

### Imagination and creativity

The contribution of the hippocampus in (re)constructing, manipulating and updating relational information extends to imaginary and future events. Neuroimaging studies have consistently shown hippocampal activation during tasks that require participants to create fictional mental scenarios, especially when they draw upon or dynamically recombine previously encoded materials (e.g., Addis et al., 2007; Buckner and Carroll, 2007; Hassabis et al., 2007a; Schacter and Addis, 2007, 2009; Schacter et al., 2007; Szpunar and McDermott, 2008; Addis and Schacter, 2008). Consistent with these data, patients with hippocampal amnesia are impaired at generating descriptions of imaginary and future events, such that their descriptions are more fragmented, contain fewer episodic and semantic details, and are poorer in overall quality than matched comparison participants (Hassabis et al., 2007b; Kwan et al., 2010; Race et al., 2013). These findings suggest that the hippocampus is required to manipulate and flexibly express stored memories into novel combinations to create the elements of imaginary events.

**Figure 1:**
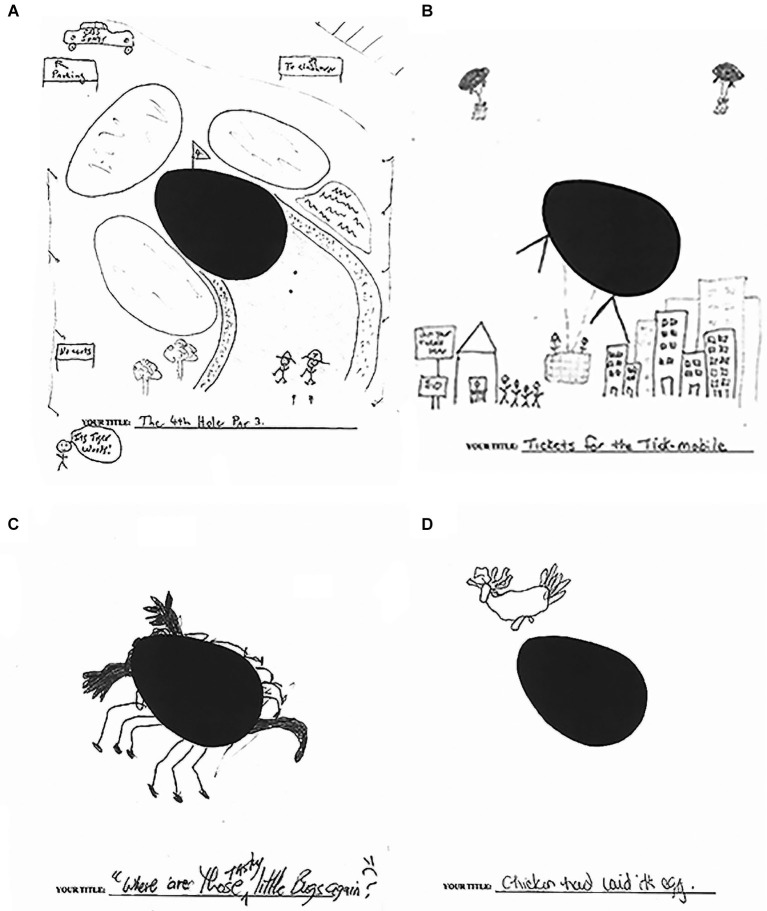
**Creativity**. Figural form example: picture construction from oval stimulus. **(A)** Comparison participant—Title: The 4th Hole Par 3; notations read from upper left clockwise: To parking; To clubhouse; Its Tiger Woods!; No carts. **(B)** Comparison participant—Title: Tickets for the Tick-mobile; notations read: Get your tickets here; $10. **(C)** Amnesic participant 1951—Title: “Where are those tasty little buggers?” **(D)** Amnesic participant 1846—Title: Chicken had laid it’s egg. (Adapted with permission from Duff et al. (2013)).

The flexibility afforded by hippocampal representations that are important for imagination also plays a critical role in the ability to engage in creative thinking more generally. Creativity requires the ability to rapidly combine and recombine existing mental representations in order to create novel ideas and ways of thinking (Damasio, 2001; Bristol and Viskontas, 2006). While cognitive flexibility is considered to be an important component of creativity and is often attributed to frontal lobe function (Dietrich, 2004; Runco, 2004; Bogousslavsky, 2005; Kowatari et al., 2009; Dietrich and Kanso, 2010), we have shown that the hippocampus is also involved in representing ideas that are important for creativity and is part of a more broadly construed creative, constructive network (Duff et al., 2013). On a well-validated, standardized measure of creativity (Torrance Tests of Creative Thinking), we found patients with hippocampal amnesia are dramatically impaired, qualitatively and quantitatively, on measures of verbal and figural creativity, relative to matched comparison participants (Duff et al., 2013). For example, on the verbal portion, participants were asked to use written language to generate creative uses for cardboard boxes during a 10 min time period. Amnesic participant 2363 produced only two responses (e.g., recycling the boxes and making a fort), while the age, education, and IQ matched comparison participant produced 26 responses, 23 of which were determined to be unique, such as building a suit of armor. We observe the same pattern on the figural portion where, on one task, participants were presented with an oval shape and asked to think of a picture that includes the oval, adding new ideas to the make the picture tell as interesting and exciting a story as possible (see Figure 1). One healthy comparison participant made the oval into a giant tick or “tick-mobile” that, similar to a hot air balloon, takes people for rides above the city. Another comparison participant used the oval as part of a golf course complete with signs for parking and the clubhouse, the CBS sports truck, and Tiger Woods with this caddy. In striking contrast, amnesic participants 1846 and 1951, despite the same stimulus and amount of time (10 min), used the oval as an egg with a chicken above it and as a bug, respectively. This deficit in creativity in amnesia is consistent with the role of the hippocampus in representational flexibility and resonates with other similar findings that demonstrate the role of the hippocampus in imagination (e.g., Addis and Schacter, 2012), making comparisons (Olsen et al., 2012), and inferential reasoning (Zeithamova et al., 2012).

### Decision-making

The role of the hippocampus in flexibly constructing and manipulating representations to imagine future possibilities and alternatives has implications for the contribution of the hippocampus in decision-making. On one such assessment, the Iowa Gambling Task (IGT, c.f. Damasio, 1994; Denburg et al., 2007), choices are associated with different amounts of rewards and punishments. In the task, participants select from four decks of cards that are overall advantageous or disadvantageous, such that some decks are associated with small rewards and also have small punishments, whereas other decks are associated with larger immediate rewards but also larger long-term punishments. The participant must learn to select from the decks that are overall rewarding. Thus, the IGT involves constructing an overall evaluation of the different decks based on integrating variable positive and negative outcomes, as well as selectively disregarding information that is inconsistent with the overall value of the deck.

These attributes make the IGT sufficiently more demanding on hippocampal representations as compared to other still complex tasks (e.g., Weather Prediction and the Wisconsin Card Sorting Task), on which patients with hippocampal amnesia perform successfully (Leng and Parkin, 1988; Janowsky et al., 1989; Shoqeirat et al., 1990; Knowlton et al., 1994). On the IGT (Gupta et al., 2009), patients with hippocampal damage differ from controls and from other patient populations. For example, patients with vmPFC and amygdala damage, known to have difficulties with real-world decision-making (Eslinger and Damasio, 1985; Stuss and Levine, 2002; Anderson et al., 2006), develop a preference for the disadvantageous decks on the IGT (Bechara et al., 1994, 1999, 2003; Fellows and Farah, 2005). Patients with hippocampal damage, however, do not develop a preference for either the advantageous or disadvantageous deck, even when there is no interposed delay after the card selection, suggesting these patients maintain only a momentary response to the outcome and employ a simplistic “lose-shift” strategy. Thus, the hippocampus is necessary to form, maintain, and update choice-outcome relations that unfolded over the course of the task, while the vmPFC and the amygdala are important to successfully integrate the information into a coherent, positive-payoff strategy. These findings are also consistent with other research in hippocampal amnesia (Gutbrod et al., 2006), patients with memory deficits resulting from Alzheimer’s type mild dementia (Sinz et al., 2008), and neuroimaging findings in healthy participants (Wimmer and Shohamy, 2012), which support the role of the hippocampus in effective decision-making.

Interestingly, one of the patients with hippocampal amnesia in Gupta et al. (2009) had more extensive bilateral MTL damage that also encompassed the amygdala. While patients with circumscribed amygdala develop a preference for the disadvantageous deck, this patient performed more like the patients with focal hippocampal damage than the patients with focal amygdala damage—failing to develop a preference for either the disadvantageous or advantageous decks. We have interpreted this additional finding to suggest that the contribution of the hippocampus may occur earlier and be more fundamental to advantageous decision-making, since patients with either amygdala or vmPFC damage are able to use hippocampal representations to develop a preference for one of the decks, albeit the disadvantageous one. Together, these findings may also explain real-world challenges that rely on similar complex decision-making abilities, such that patients with hippocampal amnesia are often unable to live independently, hold full-time employment, manage their finances, or navigate flexibility through the world.

### Character judgments

The ability to flexibly represent information afforded by the hippocampus has an important role in a range of social behaviors. The ability to learn new information about a person, or ourselves, that is tied to a specific event or experience is a characteristic feature of hippocampal-dependent memory, and contributes to our ability to form relationships with others, influences our behaviors towards others, and affects our judgments and perceptions of others. For example, hippocampal representations enable us to access multiple lines of associated information, often remote in time and space, and flexibly integrate the information with new experiences, such that the way people have behaved towards us in the past will influence the way we expect them to act in the future (Cohen and Eichenbaum, 1993; Eichenbaum and Cohen, 2001; Croft et al., 2010); however, the role of the hippocampus in social behaviors has only recently been formally investigated (with limited exceptions, e.g., Johnson et al., 1985; Tranel and Damasio, 1993; Duff et al., 2007, 2008a,b, 2009; Todorov and Olson, 2008; Croft et al., 2010; Davidson et al., 2012; Beadle et al., 2013).

**Figure 2:**
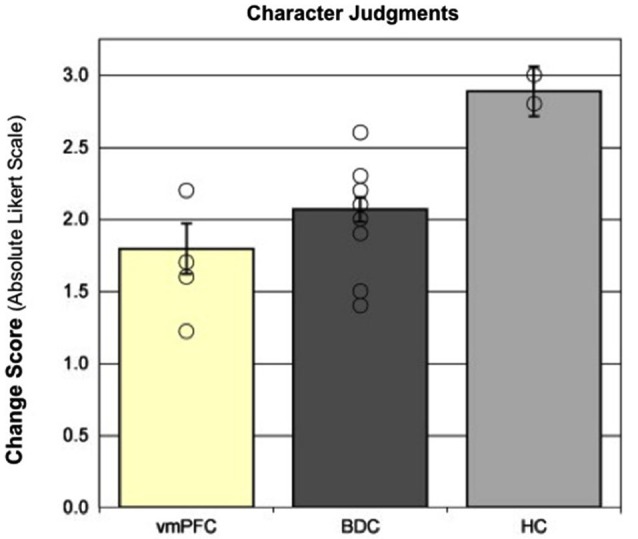
**Character judgments**. Moral updating for valenced scenarios as a function of group. This figure shows the group changes in moral judgments (in absolute Likert scale units) for morally good and bad (valenced) scenarios. Group means represent adjusted values after taking into account the effects of the covariate. Individual raw data points are plotted as open circles. Error bars represent SEM. (Adapted with permission from Croft et al. (2010)).

We investigated the contribution of the hippocampus in forming and updating character judgments by comparing the performance of patients with hippocampal amnesia to patients with damage to the vmPFC (a brain region that contributes to processing of emotional salience and moral information), as well as other brain damaged controls (Croft et al., 2010). In this study, patients made character judgments about unfamiliar persons before and after the presentation of scenarios in which a person was shown engaging in morally good, bad, or neutral behaviors. The ability to update the representation of the unfamiliar person, based on the behavior depicted in the scenario, was reflected by the change in the participant’s rating of the person (i.e., the change in the rating from before the presentation of the scenario to after the presentation of the scenario.) The patients with vmPFC damage exhibited the least change in character judgments, as expected due to their impairment in emotional processing; however, the patients with hippocampal amnesia demonstrated the greatest change and, after the presentation of the scenario, dramatically rated the persons as either very good or very bad (see Figure 2). These findings suggest that the hippocampus provides the specific contextual information from which to make appropriate character judgments, flexibly binding together information from multiple experiences, and without this signal (as well as on the IGT in Gupta et al., 2009) the patients with hippocampal amnesia overvalue the present event to make more polarized judgments.

### Social relationships and empathy

The ability to flexibly represent everyday experiences and the relations among them also impacts the capacity to form relationships with other people and maintain them overtime. Indeed, research in patients with hippocampal amnesia suggests that the hippocampus contributes to establishing and maintaining social bonds (although see Duff et al., 2008c; Davidson et al., 2012; Warren et al., 2012). In the decades since the onset of their amnesia, patients report making only a few close new friends and are less involved with neighbors, as well as religious and community groups. As a result, their social networks are significantly smaller than matched comparison participants. The inability to form, update, and flexibly deploy hippocampal-dependent memory representations likely contributes to difficultly in maintaining and developing social relationships. That is, patients with hippocampal amnesia cannot consciously recollect shared experiences, learn the names of new people, or incorporate important new information about existing relationships into their mental representations. Consistent with this perspective, performance in healthy adults on hippocampal-dependent memory tasks has been shown to predict social network size (Stiller and Dunbar, 2007).

The ability to form and maintain social relationships may also involve contributions from hippocampal representations that support the ability to imagine and reflect upon experiences with other people. That is, to consider the social relationship from another person’s perspective and exhibit empathy. Empathy is an important ability that contributes to the quality of human relationships, life satisfaction, and well-being. The cognitive and neural substrates of empathy usually include brain regions involved in processing emotional experience and perspective taking, such as vmPFC, amygdala, anterior insula, and cingulate; however, we have shown that the hippocampus is also important (Beadle et al., 2013). In this study, we measured several aspects of empathy, including perspective-taking, emotion contagion, emotional responsiveness, and empathic concern, using a variety of standard questionnaires and in response to a series of empathy inductions. Relative to matched comparison participants, patients with hippocampal amnesia reported lower cognitive and emotional trait empathy on questionnaires, and reported no increase in empathy ratings or prosocial behavior in response to empathy inductions. For example, on one of the measures of cognitive trait empathy, perspective-taking, the ratings of the patients with hippocampal amnesia were three standard deviations, or more, below that of matched comparison participants. The perspective-taking subscale of the questionnaire was designed to assess the ability of the individual to adopt the mental perspective of another person (e.g., “When I’m upset at someone, I usually try to “put myself in his shoes” for awhile”). These findings suggest that empathy places a demand on the flexible use of information, especially in terms of the ability to engage in perspective-taking, and construct and update on-line representations that incorporate new information from recent interactions—all of which involves contributions from the hippocampus. Similarly, research in healthy adults suggests that the quality of hippocampal-dependent memory representations contributes to empathic responses, in terms of facilitating the desire to endorse prosocial intentions, such that participants report increased prosocial intentions when they vividly imagine an event, or remember a past event, helping another person (Gaesser and Schacter, 2014).

### Social discourse and language use

The contribution of the hippocampus also extends to what could be considered the most complex form of flexible cognition: discourse and language use in social interaction. We have proposed that the hippocampus is a key contributor to meeting many of the demands of social discourse and language use and processing (Duff and Brown-Schmidt, 2012). Spoken language unfolds over time requiring rapid and incremental processing as multiple sources of information are generated, gathered, integrated, and maintained in real-time to create meaning. Consistent with recent accounts of hippocampal involvement over very short delays, or no delays at all, (e.g., Hannula et al., 2006; Warren et al., 2011), we have found deficits in on-line referential processing in patients with hippocampal amnesia over very short discourse histories (e.g., within and across utterances) (Rubin et al., 2011; Kurczek et al., 2013). For example, in one study we had patients view a scene while listening to short dialogues introducing two characters; for example, *Melissa is playing violin for Debbie/Danny as the sun is shining overhead. She is wearing a blue/purple dress* (Kurczek et al., 2013). Healthy comparison participants and vmPFC patients rapidly identified the intended referent of the pronoun (she) when gender uniquely identified the referent, and when it did not, they showed a preference to interpret the pronoun as referring to the first-mentioned character. Patients with hippocampal amnesia, however, while exhibiting a similar gender effect, exhibited significant disruptions in their ability to use information about which character had been mentioned first to interpret the pronoun. Findings like this, and others (e.g., Rubin et al., 2011) suggest that patients with hippocampal amnesia not only have difficulty remembering a conversation they had earlier, they also have trouble maintaining and integrating linguistic representations as they unfold over time and using that information to guide language processing in the moment.

**Figure 3:**
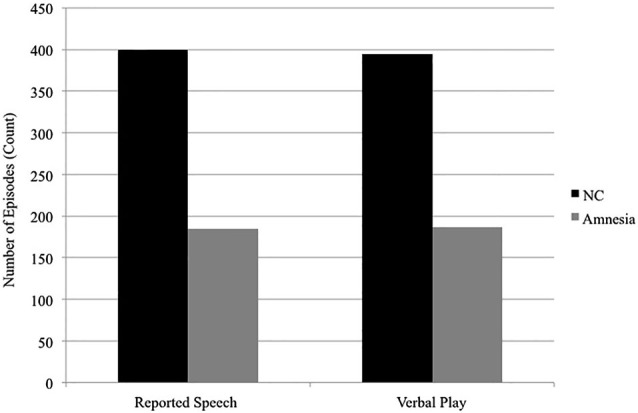
**Reported speech and verbal play.** In conversational interactions with a clinician, patients with hippocampal amnesia produce significantly fewer episodes of reported speech (185) than do normal comparisons (400). In the interactions with a familiar communication partner while completing trials of a collaborative referencing game, patients with hippocampal amnesia produced significantly fewer episodes of verbal play (187) than do normal comparisons (395). Data presented are group totals for patients with hippocampal amnesia (Amnesia) and demographically matched healthy normal comparison (NC) participants. Data of interactional partners (clinician; familiar communication partner) are not presented.

The hippocampus also contributes to social discourse, which often requires highly creative and flexible uses of language. Two examples of creative and flexible uses of language ubiquitous in social discourse include reported speech, in which speakers represent or reenact words or thoughts from other times and/or places (e.g., *If I ever have kids I’m going to tell them, please don’t say mean things to me*, Tannen, 1989) and verbal play, in which speakers play with the sounds and meanings of words through the use of puns, voices, teasing and telling funny stories (Crystal, 1998). Both of these discourse features require flexible access to previous knowledge as well as the ability to flexibly and creatively generate unique combinations of the reconstructed elements (what details to represent, what details to omit, to meet the specific interactional goals of this telling, on this occasion, with this communication partner). We have suggested that these processes are hippocampal dependent and we find deficits in the use of both reported speech and verbal play in individuals with hippocampal amnesia (Duff et al., 2007, 2009; Duff and Brown-Schmidt, 2012). That is, patients with hippocampal damage use significantly less reported speech and verbal play in their social interactions with either a clinician or familiar communication partner (see Figure 3) and when they do use reported speech or verbal play it is qualitatively different from healthy comparison participants (e.g., rotely produced). Furthermore there is some degree of hippocampal specificity; damage to vmPFC does not disrupt verbal play or reported speech (Gupta et al., 2012).

Hippocampal damage is associated with deficits across a range of linguistic and discursive abilities, although it does not, of course, affect all aspects of social discourse and language use (e.g., Gordon et al., 2014). Furthermore, hippocampal damage does not cause the devastating impairments in basic linguistic processing associated with aphasia. Yet, when the demands of social discourse and language use place sufficiently high demands on the processing features of the hippocampus, we observe deficits in the capacity to creatively and flexibility deploy the communicative and cognitive resources necessary to meet many of the moment-to-moment demands of everyday language use in social interactions.

### Reconciling accounts of the functional role of the hippocampus

The critical claim here is that representations supported by the hippocampus contribute not only to performance on memory tasks but also to a diverse set of cognitive abilities, which are engaged in accomplishing a variety of complex cognitive and social behaviors. The evidence reviewed above documents the striking deficits following disruption of the hippocampus or of its interconnections within a broad network of structures, impairing a broad array of abilities across multiple domains and paradigms. This leads to some hard questions: what is in common between the memory tasks typically associated with the hippocampus and the much broader range of abilities we now see as also dependent on the integrity of the hippocampus? And, what does this apparent expansion of the purview of the hippocampus mean with regard to accounts of the nature of the critical processing performed by the hippocampus?

On our view, the hippocampus implements a very basic functionality, but one whose reach has not always been fully appreciated: it binds together multiple items into compositional, relational representations that are stored, maintained, and updated, in the interconnections of the hippocampus with neocortical networks (more on this below), in such a way as to be available for retrieval by multiple brain processors, to be deployed *in service of* a wide variety of performances in a broad range of domains. While the functionality is basic—relational memory binding and reactivation—its reach is extensive, capable of being used *in service of* any performance that challenges or would benefit from the ability to construct, update, search, compare and contrast, and flexibly deploy relational representations across time.

Due to its capacity, in conjunction with the neocortical networks to which it is connected, to provide a rich relational database of information, the hippocampus plays an early and critical role in the formation, maintenance, and flexible deployment of representations that are then used by other neural systems *in service of* flexible cognition and complex social behavior. More specifically, we propose as an individual navigates through dynamic spatial and social environments in the world, the hippocampus is creating rich relational representations of the present while simultaneously and automatically recovering previous experiences that are similar in content and/or context and may generate novel scenarios of possible future events and outcomes (see Eichenbaum and Cohen, 2014; Wang et al., 2014). The flexibility afforded by these hippocampal representations allows them to be made immediately available to other neural systems promiscuously (Cohen, 1984). Thus, other structures in the network specialized for weighting social information and social decision-making (e.g., PFC and amygdala) can then use the information (i.e., representations of past, present, and possible future(s)) made available by the hippocampus to make decisions about the best outcome or course of action. To highlight just one example emphasizing the critical role of the hippocampus within a larger brain network, recall from the studies using the IGT that the hippocampus is necessary to form, maintain, and update choice-outcome relations that unfolded over the course of the task, while the vmPFC and the amygdala are critical to successfully integrate the information into a coherent, positive-payoff strategy.

The functionality described here is fully consistent with our previous accounts of the hippocampus (e.g., Cohen and Eichenbaum, 1993; Eichenbaum and Cohen, 2001, 2014; Eichenbaum, 2004; Wang et al., 2014). The emphasis here is on the idea that hippocampal function is not limited to particular domains of memory (e.g., just space or just explicit remembering), and not limited only to the domain of memory, but instead is available to and in service of any aspects of behavior or performance that place high demands on the formation or use of relational and flexible representations. This idea is also consistent with points of emphasis of other contemporary investigators, including ideas about the role of the hippocampus in scene (re)construction (e.g., Maguire and Mullally, 2013), and in the generation of simulated future events (Buckner and Carroll, 2007), as well as the functional relationship between the hippocampus and the PFC in service of episodic memory (Ranganath, 2010b). Moscovitch (2008) has also talked about the hippocampus in ways reminiscent of our views on relational memory binding, and about the critical interaction of hippocampus with the PFC. He emphasizes that the hippocampus is a “stupid” module, and attributes to the PFC subsequent and ostensibly more important processing of the information, with ultimate decision- and choice-making (Moscovitch, 2008). We would emphasize that the extent that the hippocampus successfully forms and recovers all the pertinent information of the past, present, and possible future options, in addition to maintaining that information online and making it available to other neural systems, has a profound influence on and plays a critical role in the outcome of what neural systems can accomplish. This is true not just for memory performance, narrowly, but as we have documented here, also for flexible cognition and social behavior more generally. So, while the hippocampus may not make decisions about what pieces of information to encode or make decisions about how to act on those representations, its binding, construction, updating, and reactivation of relational representations are critical to flexible cognition and social behavior.

A different thread in the current literature on memory and hippocampus is less focused on the interconnections of the hippocampus and larger brain networks, and more focused on the internal structure of, or anatomical subdivisions within, the hippocampus, emphasizing the memory-related sub-processes of pattern separation and pattern completion (e.g., Norman and O’Reilly, 2003; Bakker et al., 2008). But, here, too, such memory operations are thought to be applicable to all kinds of information, contributing to the creation and retrieval of the kinds of representations that we suggest here have such a broad impact on flexible cognition and social behavior.

## The hippocampus is anatomically connected to brain regions known to support flexible cognition and social behavior

The role of the hippocampus in flexible cognition and social behavior is further revealed by the neuroanatomical and functional connections between the hippocampus and other brain structures. While the hippocampus is extensively connected with surrounding MTL structures, including the entorhinal, the perirhinal, and the parahippocampal cortices, we focus here on the connectivity between hippocampus and brain structures that are traditionally thought to be involved in executive function and social interactions, such as the PFC, the amygdala, and the cingulate (Simons and Spiers, 2003; Wood and Grafman, 2003). Both neuropsychological patient and functional neuroimaging studies have demonstrated the involvement of the PFC in complex abilities that require flexible cognition, such as moral reasoning, social conduct, experiencing and recognition of social emotions, assigning affective value to mental representations, and social and emotional decision-making (e.g., Eslinger and Damasio, 1985; Damasio et al., 1990, 1991; Bechara et al., 1994, 1997; Anderson et al., 1999; Greene et al., 2001; Gusnard et al., 2001; Berthoz et al., 2002; Gregory et al., 2002; Shuren and Grafman, 2002; Stuss and Levine, 2002; Bar-On et al., 2003; Beer et al., 2003; Frith and Frith, 2003; Sabbagh, 2004; Mah et al., 2005; Moll et al., 2005; Hynes et al., 2006; D’Argembeau et al., 2008). The hippocampus has extensive connections with the PFC (Simons and Spiers, 2003; Wood and Grafman, 2003), including direct reciprocal connections between the medial PFC and the MTL (Simons and Spiers, 2003), reciprocal connections between the PFC and the perirhinal cortex (Lavenex and Amaral, 2000), unidirectional projections from the hippocampus to the vmPFC (Rosene and Van Hoesen, 1977; Barbas and Blatt, 1995), and bidirectional connections from the subiculum and neocortical medial temporal regions to the vmPFC (Goldman-Rakic et al., 1984; Barbas et al., 1999).

The hippocampus is also extensively involved in the limbic circuit, with extensive connectivity between the amygdala and the cingulate. Many research findings have documented the role of the amygdala social and emotional behavior. The amygdala is important for the detection and recognition of emotional facial expressions (Vuilleumier et al., 2001; Adolphs, 2002, 2003; Adolphs et al., 2005), for the processing of social information more generally (e.g., Hariri et al., 2002; Norris et al., 2004), for advantageous complex decision-making (Bechara et al., 1999), for taking the perspective of others (Baron-Cohen et al., 1999; Stone et al., 2003), and for fear conditioning (e.g., LeDoux, 2003). Furthermore, there is clear evidence that the amygdala is critical for the enhancement of declarative memory by emotion (Bradley et al., 1992; Buchanan and Adolphs, 2002, 2004; McGaugh, 2004; Phelps, 2004; Adolphs et al., 2005; LaBar and Cabeza, 2006). Similarly, the cingulate is involved in both reward processing (Hadland et al., 2003) and emotional memory (Frankland et al., 2004), seemingly by transmitting and elaborating information passing between the hippocampal system and neocortical association areas (Sutherland et al., 1988). Functional neuroimaging studies have also demonstrated correlated activation in the amygdala and the PFC (Rilling et al., 2002; Greenberg et al., 2005), correlated activation of the amygdala and the hippocampus for emotional pictures (Dolcos et al., 2004; Richardson et al., 2004), and prefrontal-cingulate networks in emotional processing (Etkin et al., 2011) and decision-making (Elliott and Dolan, 1998; Rogers et al., 2004).

The hippocampus is anatomically connected to the amygdala via the basal nucleus, the accessory basal nucleus, and the lateral nucleus (Pikkarainen et al., 1999). Furthermore, the amygdala is connected bidirectionally to the PFC, especially the medial aspects, directly and via the dorsomedial thalamus (Bachevalier, 2000; Öngür and Price, 2000). With regards to the cingulate, the hippocampal formation sends dense projections to the anterior cingulate gyrus (Wyass and Van Groen, 1992) and the posterior cingulate cortex receives direct afferents from the subiculum of the hippocampus (Adey, 1951).

Thus, the hippocampus is both neuroanatomically and functionally connected with brain structures that are important for decision-making, adaptive reasoning, executive function, and social behavior, emphasizing the contribution of the hippocampus to an extensive network of brain structures that enable us to engage in successful and adaptive behavior. Of course, flexible cognition requires the orchestration of the full network, yet performance of patients with focal hippocampal and vmPFC damage suggests that distinct neural systems may differ in the nature and timing of their contribution (e.g., patients with hippocampal damage and vmPFC damage show different patterns of deficit on the IGT task and on the character updating task). Delineating the nature and time-course of the interactions between hippocampus and the rest of the network for flexible cognition promises to offer finer-grained understandings of these complex dynamics. Development of tasks that are sufficiently complex to recruit diverse neural systems and data analyses sufficiently sensitive to detect the timing and contribution of individual systems will also further our understanding of how the network as a whole operates in real time and in complex environments in service of adaptive and social behavior. Indeed, even patients with hippocampal damage can often rely upon perception and prior semantic knowledge to guide their behavior in many circumstances, causing investigators to underestimate all the ways in which such patients might be impaired if properly challenged.

## Translating advances in basic cognitive neuroscience into clinical applications

Converging evidence shows that varying degrees of hippocampal dysfunction have been implicated in a wide variety of patients with neurological conditions, such as traumatic brain injury and Alzheimer’s disease, as well as psychiatric conditions, such as schizophrenia, post-traumatic stress disorder (PTSD), depression, anxiety, and autism (Heckers et al., 1998; Nelson et al., 1998; Campbell and MacQueen, 2004; Schumann et al., 2004; Shin et al., 2006; Etkin and Wager, 2007). For example, in schizophrenia patients we found the same kind of impairment on a hippocampal-dependent relational memory paradigm (Williams et al., 2010), though to a lesser degree, than amnesic patients with profound hippocampal damage (Hannula et al., 2007). In depressed patients, structural neuroimaging studies revealed reduced hippocampal volumes relative to control groups (Bremner et al., 2000; Mervaala et al., 2000; but see Posener et al., 2003). In patients with PTSD, a meta-analysis of functional neuroimaging studies found hypoactivations during fear-conditioning in a network of structures, including the anterior hippocampus, relative to a control group (Etkin and Wager, 2007). The hippocampus also plays a role in regulating the hypothalamic-pituitary-adrenal axis (Fendler et al., 1961; Jacobson and Sapolsky, 1991), which is perturbed in both PTSD (Buckley et al., 2000; de Kloet et al., 2006) and depression (MacQueen et al., 2003; Heim et al., 2008). Thus, the evidence that hippocampal dysfunction can be found in clinical cases more broadly than only those discussed in the amnesia literature raises an interesting question as to whether clinicians may be more aware of the larger reach of the hippocampus, beyond its impact on traditional memory test performance, than we get from the amnesia literature alone.

Once we understand that hippocampal insult (whether focal and primary or secondary as part of a more diffuse pathology) is disruptive to the formation and use of flexible representations in service of flexible cognition, we see how it can underlie deficits in the seemingly distinct domains of memory, language, social interaction, etc. Thus, the patient with hippocampal disruption who is unable to integrate knowledge during a complex task, use specific details to plan a future event, track the status of a social interaction over time, and, more generally, reach outside the contents of their current experience, will surely exhibit broader disruptions of everyday life. These disruptions will be manifested in their social behaviors, shrinking the range and quality of their social interactions, and in their decision-making, resulting in taking on fewer day-to-day responsibilities and more difficulty with everyday activities, much of which is likely to be seen by clinicians in their interactions with these patients. We suggest that these various deficits or changes in everyday life for such patients emerge from a common cause, namely the deficit in formation and use of flexible representations.

This view of how a deficit in what is classically seen as limited to the domain of memory actually extends across many domains of cognition is, intriguingly, very much in line with the NIMH’s recently created Research Domain Criteria (RDoC) project, where researchers are encouraged to shift from focusing on categorically distinct mental disorders, such as schizophrenia or major affective disorder, to instead focus on underlying symptoms, or disruptions of dimensions of cognition and behavior, such as depression or hallucinations, that might cut across disease categories. Here, we are arguing that declarative memory, one of the dimensions described in RDoC, when impaired, causes deficits that extend across a range of cognitive domains and impair or disrupt behavior in a wide range of neurological and psychiatric conditions. Moreover, consistent with the RDoC focus on underlying brain systems and brain mechanisms, our consideration here of declarative memory, and its various manifestations, is tied squarely to the hippocampus and the brain networks with which it interacts.

## Conclusion

Humans interact with and actively participate in the world around them. The ability to make sense of the events of daily life, and to act optimally in and on the world requires the constant creation, modification, and use of flexible representations. The ability to flexibly manipulate, update, and integrate information is essential, allowing us to blend past experiences with future goals to make appropriate decisions. The findings reviewed here demonstrate that the hippocampus plays a critical role in flexibly representing information important for many aspects of cognition and social behavior. The hippocampus supports the ability to bind and flexibly represent discrete elements of an experience and, through its interconnections with other neural systems, permits the expression of flexible and adaptive behavior. Together, these findings also highlight the unique perspective that research in patient populations provides, when investigating the contribution of a specific brain structure to a variety of complex behaviors, and the translational value of such research to clinical practice. The flexible cognitive and social abilities reviewed here are required to successfully engage in everyday activities; however, only recently has the hippocampus been recognized as one of the brain structures important for flexible and adaptive human interactions, which is related to, but beyond its traditionally recognized role in memory.

## Conflict of interest statement

The authors declare that the research was conducted in the absence of any commercial or financial relationships that could be construed as a potential conflict of interest.
